# Closed reduction of severely displaced radial neck fractures in children

**DOI:** 10.1186/s12891-019-2947-8

**Published:** 2019-11-27

**Authors:** Fei Qiao, Fei Jiang

**Affiliations:** Department of Pediatric Orthopaedic, Dalian Children’s Hospital, 154 Zhongshan Road, Dalian, 116012 China

**Keywords:** Radial neck fracture, Percutaneous leverage, Intramedullary pinning

## Abstract

**Background:**

Severely displaced radial neck fractures in skeletally immature children are rare and can be difficult to reduce. The purpose of this study is to evaluate the results using our reduction maneuver.

**Methods:**

From October 2011 to December 2015, 26 children with radial neck fractures(O’Brien type II, III and Judet type III, IV) were treated at our institute. All patients underwent percutaneous K-wire leverage and radial intramedullary pinning in an average surgery time of 35 mins (15–80 min). The injured arm was immobilized at the functional position with plaster for 4–6 weeks, evaluated clinically and radiologically. The Metaizeau classification and Mayo elbow performance score were used to evaluate the radiological and clinical results, respectively. Percutaneous K-wire leverage and radial intramedullary pinning were performed for 26 patients. No patients were treated with open reduction. Twenty four patients were followed up for a mean of 33 (range 12–53 months) months.

**Results:**

There were 15 girls and 9 boys with ages ranging from 1.5 to 12 years and an average age of 7.2 years. Percutaneous K-wire leverage reduction and intramedullary pinning were successfully used in an average total surgery time of 35 mins (range 15–80 min). In total, 2 cases (O’Brien type III and Judet type IVb, angulation = 90°) needed the additional maneuver. Bone union was achieved in all patients within a mean time of 4.2 weeks. The clinical results were evaluated basing on the Mayo elbow performance score, and there were 23 excellent results and one good result. There were no refractures and no incidences of nonunion, suture infection, iatrogenic radial nerve injury, asymptomatic enlargements of the radial head or growth arrest in the proximal radial epiphysis.

**Conclusion:**

Our modified percutaneous leverage technique with radial intramedullary fixation may be successfully used to avoid open reduction.

## Introduction

Radial neck fracture accounts for 4.5 to 21% of pediatric elbow fractures. Most radial neck fractures are minimally displaced or nondisplaced [1–3]. Elbow fractures often occur as a result of falling onto an outstretched hand with elbow in extension [4]. The vast majority of radial neck fractures which are undisplaced or minimally displaced, can be treated nonoperatively with good outcomes, especially for young patients with an angulation less than 30° [2]. Most authors have advocated for closed reduction for fractures with an angulation greater than 30°, although there have been recommendations for closed reduction for angulations ranging from 20° to 45° of the initial angulation [5]. These fractures are classified according to the O’Brien [6] and Judet [7] classification, which have been suggested to be the both effective guides in both treatment and prognosis. Severely angulated fractures (O’Brien type II, III and Judet type III, IV) are rare and require closed or open reduction and internal fixation, which can include several techniques [8, 9]. Percutaneous leverage reduction and internal fixation techniques for severely displaced radial neck fractures have been further developed since the first description by Feray in 1969 and provide a minimally invasive procedure than other techniques [10]. Since 1993, closed extracapsular reduction by the Metaizeau technique has gained excellent results but can result in complications such as proximal radial physeal injury, damage of extensor pollicis longus and injury of sensory radial nerve at the insertion site of the pin tail [4, 11–13].. This article is a retrospective study of our experience treating radial neck fractures (O’Brien type II, III and Judet type III, IV) using a modified percutaneous leverage technique and radial intramedullary fixation.

## Methods

### Patients

This study was approved by the Institutional Ethical Review Board of Dalian Children’s Hospital, (approval number 007–2018). Written informed consent was obtained from all guardians for anonymized data analysis and publication. A total of 26 children with radial neck fractures (O’Brien type II, III and Judet type III, IV) were treated at our hospital from October 2011 to December 2015. A total of 24 patients were followed up for a mean of 33 months (range 12-53 months). Eight cases were classified as O’Brien type II and Judet type III (mean angle 41.11°; range 35°-55°), and 16 cases were classified as O’Brien type III and Judet type IV (mean angle 81.47°; range 65°–90°). There were 9 boys and 15 girls with a mean age of 7.2 years (1.5–12 years). The fractures were on the right side in 7 patients and on the left side in 17 patients. All fractures were closed reductions with no associated vascular or neuronal injuries. All procedures were performed by the senior surgeon in an average surgery time of 35 mins (range 15–80 min). Five patients were complicated with proximal ulnar fractures; 1 patient had a lateral condyle humerus fracture, and 2 patients had radial nerve injuries, all of whom recovered within 3 and 5 months after the operation, respectively. The mean time from injury to surgery was 1.3 days (0.5–3 days). The injured arm was immobilized at the functional position with plaster for 4 to 6 weeks.

### Surgical procedures

**Fig. 1:**
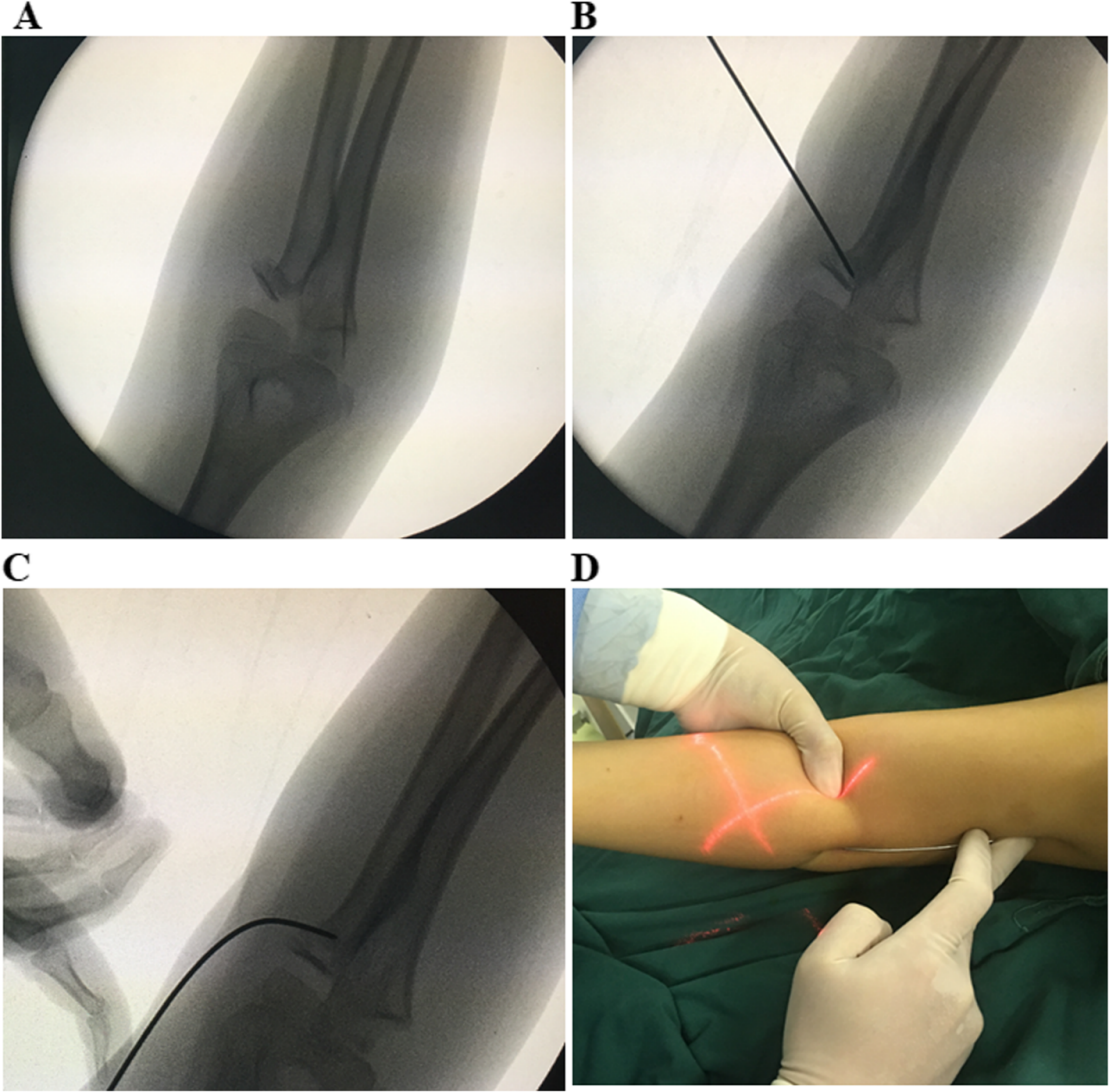
**a** An O’Brien type II fracture in a patient. **b** The K-wire was percutaneously inserted and leveraged before the intramedullary nail was inserted into the patient. **c** The leverage procedure was performed to reduce the fracture in the patient before the intramedullary nail was inserted. **d** The additional reduction maneuver for the initially unsuccessful reductions, with patient No.20 as an example

**Fig. 2:**
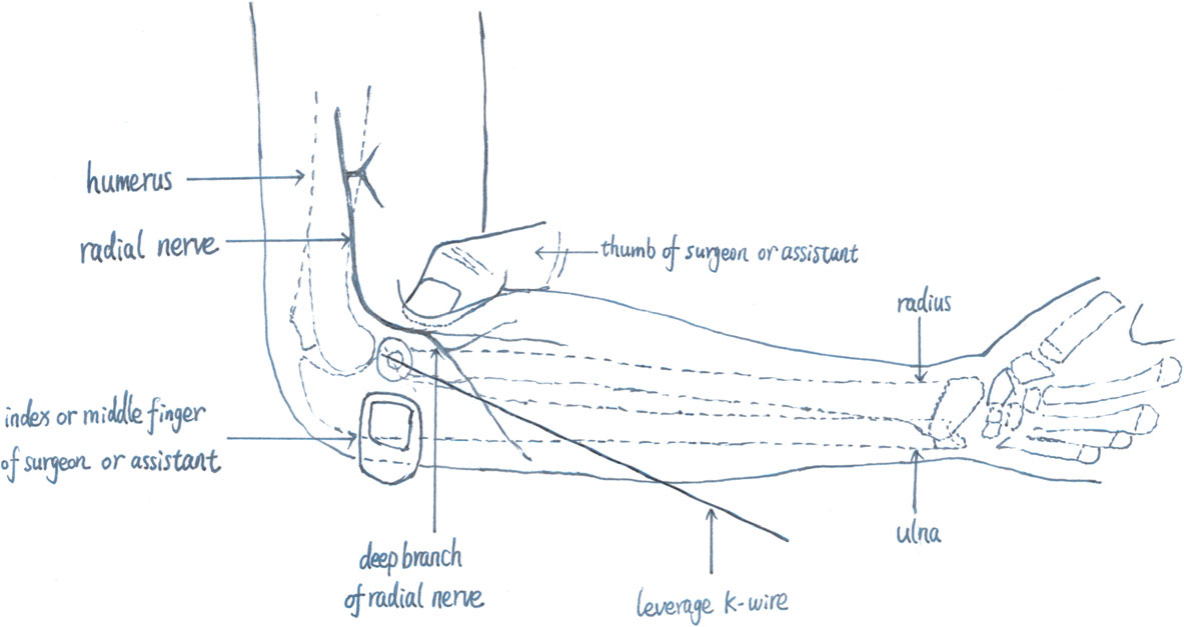
Diagram of our additional reduction maneuver for leverage

**Fig. 3:**
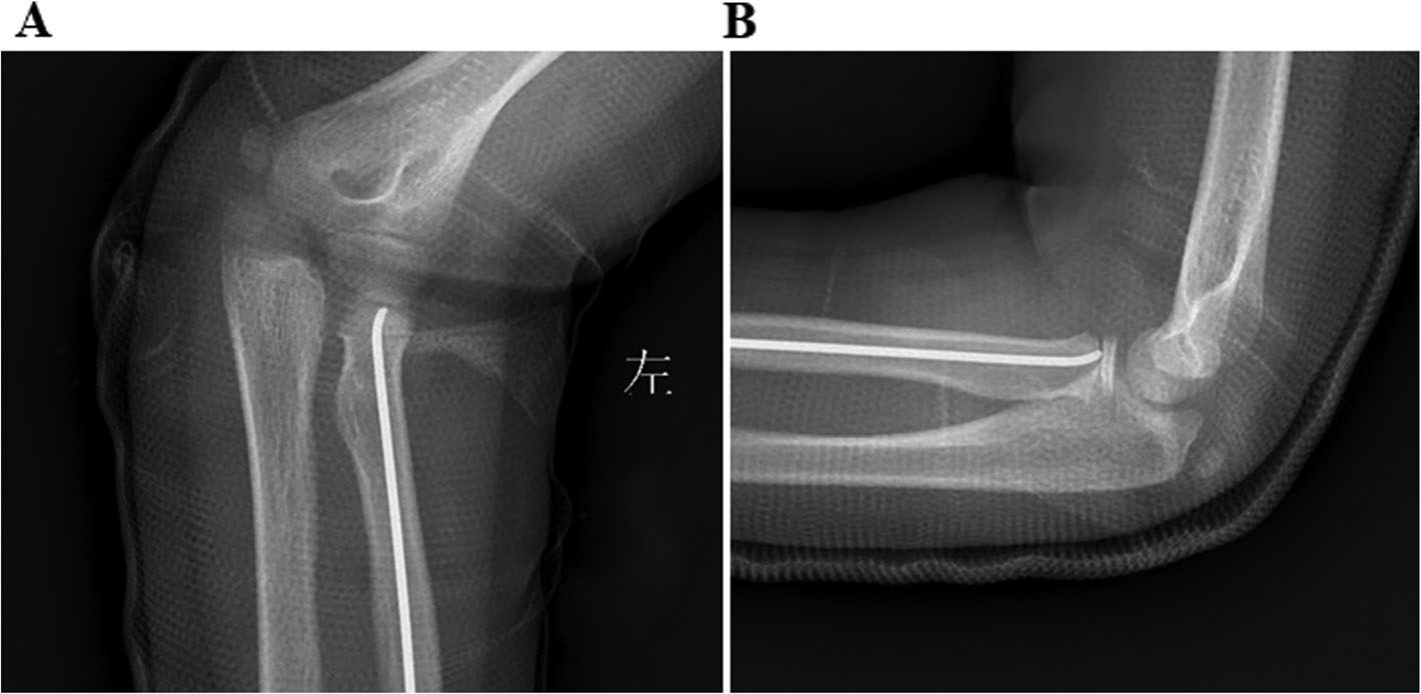
Postoperative AP and lateral X-ray of patient No. 20

All patients received general anesthesia. First, under the guide of the C-arm image intensifier, a leverage K-wire with a 2.0 mm diameter was percutaneously inserted into the bone fragment from the displacement direction of the fractured radial neck fragment. Reduction of the fracture was achieved by leveraging the K-wire and through manual reduction. Then reduction was confirmed with an image intensifier (Fig. 1a, b, c). If the reduction failed, the proximal fracture portion moved dorsally and ventrally after several manipulations, one additional maneuver followed. Keeping the injured elbow flexion and neutral position, we let the assistant or surgeon himself with their thumb and index or middle finger to clamp toughly the anterior and posterior of the space between the distal fracture and capitellum to prevent movement in the proximal fracture portion (Fig. 1d and Fig. 2). Then, leverage was conducted again to achieve fracture reduction. After the percutaneous K-wire leverage, an elastic intramedullary nail with a diameter 0.7 times the narrowest site of the radial bone marrow cavity was used. The nail was inserted into the radius 1.0–2.0 cm from the proximal side of the distal epiphyseal plate to ensure no iatrogenic neurovascular injury and was advanced proximally to the fracture site. The intramedullary nail was inserted proximal to the radial epiphysis and advanced to the fracture site and fixed (Fig. 3). All radial neck fractures were reduced to < 30° and a displacement < 30%. After successful reduction, the excessive part of the nail was bent to an angle of 45°. A 5 mm length of the nail was reserved outside of the bone. The injured arm was fixed at the functional position with plaster for 4 to 6 weeks. Exercise was encouraged after the removal of the plaster.

### Postoperative evaluation

The first clinical and radiographic review was conducted 2 weeks after surgery. Then, the fixation and bone union were radiographically examined at 4 weeks, 6 weeks, 8 weeks, 3 months, and 6 months postoperatively and thereafter at half-year intervals. The bone union was indicated by the disappearance of fracture lines in 3 of the 4 cortices on both the antero-posterior and lateral radiographs of the elbow. Clinical control was achieved 4 or 6 weeks after surgery with the removal of the cast, and continuous passive motion (CPM) was started after the removal of the plaster to recover the full range of motion of the injured elbow. The nail was removed after at least 3 months when there was radiological healing. The average follow-up period was 33 months (range 12–53 months). The results were assessed radiologically using the Metaizeau classification [4] (Table 1) and clinically by the Mayo elbow performance score .

**Table 1:** Metaizeau classification

Result	Description(anagulation in A-P)
Excellent	Anatomic reduction
Good	<20degrees
Fair	20-40degrees
Poor	> 40 degrees

### Statistical analysis

Statistical analyses were undertaken using SPSS v22. When the distribution was nonparametric, a Mann-Whitney U test for independent samples was performed. The Paired-Samples T test was used to analyze the difference between the injured and contralateral elbow when the data was parametric. Significance was set at *p* < 0.05.

## Results

**Fig. 4:**
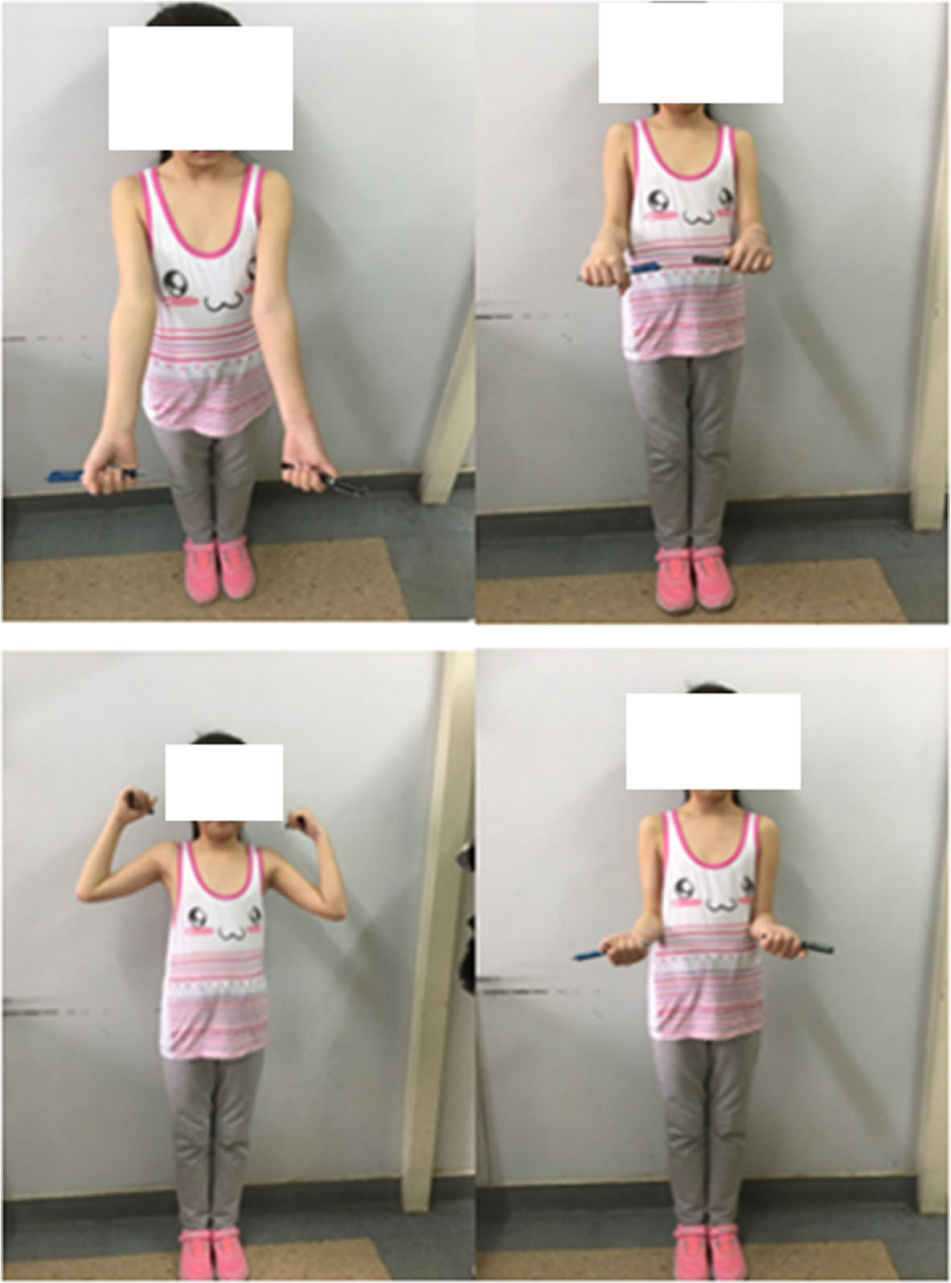
Mayo score of patient No. 6

**Fig. 5:**
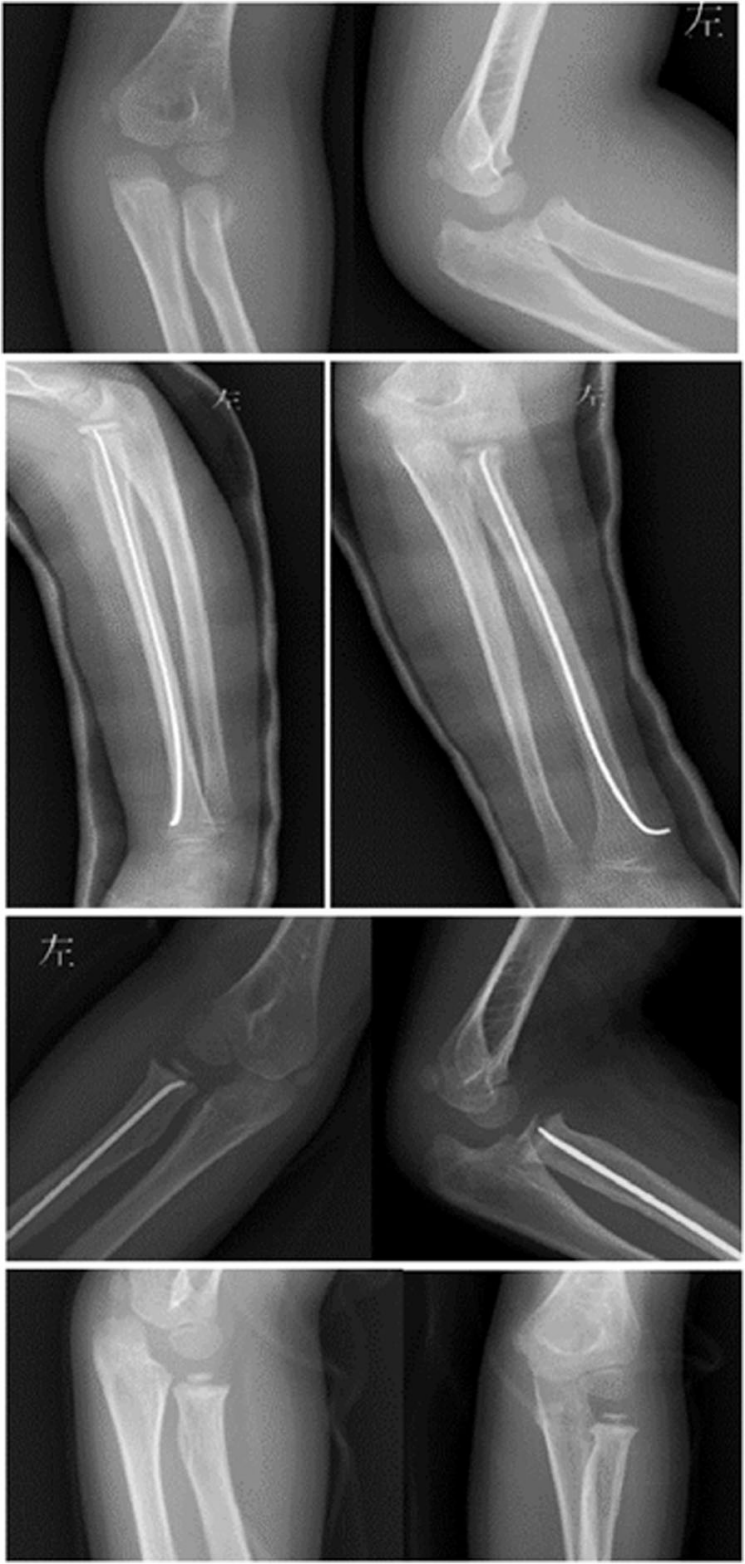
preoperative x-ray, postoperative and final follow-up X-rays of patient No. 6

There were 15 girls and 9 boys with ages ranging from 1.5 to 12 years and an average age of 7.2 years. Left-sided involvement was observed in 17 (70.83%) patients, and right-sided involvement was observed in 7 (29.17%) patients. Eight cases were classified as O’Brien type II and Judet type III (mean angle 41.11°; range 35°-55°), and 16 cases were classified as O’Brien type III and Judet type IV (mean angle 81.45°; range 65°–90°). Percutaneous K-wire leverage reduction and intramedullary pinning were successfully used in an average total surgery time of 35 mins (range 15–80 min). In total, 2 cases (O’Brien type III and Judet type IVb, angulation = 90°) needed the additional maneuver. Bone union was achieved in all patients within a mean time of 4.2 weeks. The postoperative radiographic controls evaluated assessed by the Metaizeau score showed that the quality of the reduction was excellent in 21 patients and good in 3 patients (Table 2). The clinical results were assessed by the Mayo elbow performance score, and there were 23 excellent results and one good result. There were no refractures and no incidences of nonunion, suture infection, heterotopic ossification, osteomyelitis, iatrogenic radial nerve injury, radioulnar synostosis, asymptomatic enlargements of the radial head or growth arrest in the proximal radial epiphysis (Figs. 4 and 5, patient No. 6).

**Table 2:** Patients, classification, and outcomes

Patient	Age(yrs)	Gender	Side	Length of op(mins)	O’Brien/Judet classification	Angulation pre-op(degree)	Follow-up(months)	Metaizeau classification	Mayo score
1	7	f	L	30	III/IVb	90	34	Excellent	Excellent
2	12	m	L	35	III/IVa	80	42	Excellent	Excellent
3	12	f	L	15	III/IVa	65	34	Excellent	Excellent
4	12	m	L	20	III/IVa	75	50	Excellent	Excellent
5	9	m	L	30	III/IVb	90	45	Good	Excellent
6	11	m	L	50	III/IVb	90	45	Excellent	Excellent
7	8	m	L	30	III/IVa	75	lost	Excellent	Lost
8	10	m	L	50	III/IVb	85	12	Good	Good
9	9	f	L	80	III/IVa	75	53	Excellent	Excellent
10	9	f	L	30	III/IVb	90	18	Excellent	Excellent
11	5	f	L	35	III/IVa	80	17	Excellent	Excellent
12	5	f	L	40	III/IVa	70	16	Excellent	Excellent
13	8	m	L	40	III/IVa	80	33	Excellent	Excellent
14	8	m	L	15	III/IVb	90	20	Good	Excellent
15	6	m	R	20	III/IVb	90	22	Excellent	Excellent
16	3	f	R	80	III/IVa	70	52	Excellent	Excellent
17	8	f	R	50	III/IVb	90	34	Excellent	Excellent
18	8	m	L	30	II/III	35	42	Excellent	Excellent
19	1.5	f	L	25	II/III	35	52	Excellent	Excellent
20	9	f	L	30	II/III	40	41	Excellent	Excellent
21	7	f	L	25	II/III	40	33	Excellent	Excellent
22	3	f	R	20	II/III	45	lost	Excellent	Lost
23	4	f	R	35	II/III	55	32	Excellent	Excellent
24	6	f	R	45	II/III	35	21	Excellent	Excellent
25	2.5	f	R	20	II/III	45	19	Excellent	Excellent
26	3	f	R	35	II/III	40	24	Excellent	Excellent

## Discussion

The management of obviously displaced radial neck fractures in children remains a challenge in pediatric orthopedics. There is a general agreement that conservatively treated moderate and severe displaced radial neck fractures with an angulation > 30° can result in a decreased range of motion (ROM) and increase the risk of avascular necrosis [10, 14–16]. A series of surgical procedures have been reported, including percutaneous or intramedullary fixation and open or closed reduction. Open procedure has been conventionally recommended for unsuccessful closed manipulation in this kind of fractures but leads to bad outcomes [1, 14, 17]. Close reduction achieves better clinical results than open procedure. In severely angulated radial neck fractures, closed reduction alone without fixation has unpredictable results ranging from inability to achieve complete reduction to even loss of reduction inside the plaster [18, 19].

In 1993, Metaizeau et al. reported intramedullary nailing as a surgical option for the treatment of displaced radial neck fractures [4]. The main superiority of intramedullary fixation is that it simultaneously allows for accurate and stable reduction without disturbing the blood supply [20]. However, this treatment must be performed carefully to protect the superficial branch of the radial nerve and the radial physis [21]. Intramedullary nailing has acceptable indirect reduction and preserves the lateral periosteum and the epiphyseal vascular supply; both of these are associated with internal fixation, which prevents displacement before fracture healing [22]. Moreover, several authors have reported excellent results with percutaneous K-wire manipulation; however, they still recommended that this technique may not be used for radial neck fractures with major translocation [10, 17, 23–26].

A research compared the results in patients with severely displaced radial neck fractures treated with ESIN and percutaneous pinning techniques. Both methods achieved excellent results. However, the ESIN technique seems to be the better approach [22]. In our study, there was no complications such as nonunion, growth arrest in the proximal radial epiphysis, radioulnar synostosis, iatrogenic nerve injury and periarticular ossification and asymptomatic enlargements of the radial head. The minor complication of inadvertent radial head inversion during closed reduction reported by Sirois [27] wasn’t represented in our cases.

From our cases, one 1.5 years old child with a displaced fracture of radial neck (O’Brien type II and Judet type III) may be difficult to monitor the reduction for the unossified radial neck, the only clue was irregularity in the smoothness of proximal metaphyseal margin. During closed reduction, the metaphyseal margin leads the leverage under fluoroscopy. The use of arthrogram and ultrasonography to assess the extent of displacement and the accuracy of reduction would be good choices in children with unossified radial head [28, 29]. According to Salter-Harris (SH) classification two groups were composed as follow: group A 15(62.5%) SH- type I or II and group B 9 (375%) total metaphyseal fracture [30]. The different type of fractures did not affect the duration time of reduction procedures significantly (group A mean 0.24 min vs group B mean 0.30 min, *p* > 0.05).

In our study, most of the duration time of reduction procedures were performed within 0.29 min by 2–3 attempts of leverage, but 2 cases (O’Brien type III and Judet type IVb, angulation = 90°) required an especially long time to manage the leverage reduction. The initial unsuccessful K-wire leverage occurred when the proximal fracture portion moved dorsally and ventrally after several manipulations. However, our additional maneuver provided a second chance for the initially unsuccessful K-wire leverage to avoid open reduction. The ratio of open reduction of severely displaced radial neck fractures was ranged from 6.2 to 38.5% reported before [7, 11, 17], but in our cases we achieved 100% closed reduction. Even for the 2 initial leverage failed cases, with the introduction of our additional maneuver, closed reductions managed. Our experience will be the excellent supplement of closed reduction maneuver of this kind of fractures.

Generally, higher closed reduction rates are associated with more radiation exposure (RE). RE is associated with leukemia, solid organ and thyroid cancer [31]. The risk of RE need to be understood and minimized in pediatric trauma theatres. Recent studies of RE in elbow fracture of children reported that the mean DAP (dose area product) were ranged from 22.3 to 87.41 mGy/cm2. The lower screening times and RE was found in procedures performed by consultant and senior register [32, 33]. One study documented that the equivalent dose to the thyroid and gonads of patients was minimal and approximates daily background radiation with lead shield during operative fixation of pediatric supracondylar humerus [34]. Ultrasonography (US) has also been reported using intraoperative guidance for the reduction of radial neck fractures in children. The use of US can identify the pin for reduction and constantly monitor the reduction in multiple planes. However, US can identify only the near cortex of bone [28]. From our own experience, we recommend the limits to fluoroscopy time by several methods: (1) Avoiding repeated or redundant images; (2) These procedures should either be operated or be supervised by senior surgeons; (3) Surgeons should be familiar with the anatomic landmarks and palpation of fractures to minimize the using of C-arm intensifier.

In previous studies, most surgeons agreed that closed reduction might fail in severely displaced fractures [1, 20, 21]. However, few of those authors analyzed why the closed reduction failed and how closed reduction can be achieved for the failed cases. Nitin Bither et al. recommended the presence of a periosteal hinge as an obvious marker of success in closed reduction [24]. When we reviewed the unsuccessful procedures, we hypothesized that the integrity of the lateral periosteal hinge and elbow capsule is the anatomic basis of a successful closed reduction, especially in O’Brien type III and Judet type IVb fractures with angulation = 90°and severe edema for which the initial percutaneous leverage failed.

We preferred the percutaneous K-wire leverage to the Metaizeau method for two reasons. First, our reduction maneuver minimize iatrogenic insults to the fragile proximal radius. The potential insults are cumulative trauma from repeated failed manipulation reductions [2, 9]. Higher fracture angulation and increased displacement (more severe O’Brien type and Judet type) were associated with more invasive interventions in the cases reported by Zimmerman et al. [35]. Second, the radial neck is vascularized by the branches of the radial recurrent artery and the branch ulnaris of the ulnar artery [36]. The protection of these two artery structures is critical to fracture healing. During the reduction procedure in the Metaizeau method, the rotation of the periosteal hinge may tear the residual proximal radius to damage the blood supply, which is critical to fracture healing. While passing the 2.0 mm K-wire through the fracture, the surgeon must be careful not to resist resistance to protect the annular ligament [25]. Percutaneous K-wire manipulation may lead to damage to the physis. However, in our study, we did not observe any damage when the lever arm technique was used. We also performed the technique carefully to avoid injury to the sensory branch of the radial nerve and distal radial physis.

## Conclusions

Our modified percutaneous leverage technique with radial intramedullary fixation may be successfully used to avoid open reduction. This technique is simple and minimally invasive, and the results are excellent. In our opinion, the integrity of the lateral periosteal hinge and elbow capsule is a key indicator for a successful closed reduction, especially in O’Brien type III and Judet type IVb fractures for which the initial percutaneous reduction failed and therefore, through our experience, should be treated with our reduction maneuver.

## Data Availability

All data generated and/or analyzed during the current study are available in this published article. Data required that are not in the article are available from the corresponding author on reasonable request.

## Abbreviations

CPM: Continuous passive motion
RE: Radiation exposure
ROM: Range of motion
US: Ultrasonography
